# Three stepping stones leading to malaria elimination, changing world maps on the way

**Published:** 2020-01-01

**Authors:** Florence V. Dunkel, Anton Alexander

**Affiliations:** 1 Department of Plant Sciences and Plant Pathology, Montana State University, Bozeman, MT 50717 USA;; 2 BC Business Centrum, Elscot House, Arcadia Avenue, London N3 2JU, UK.

## Abstract

Over the course of human history, malaria has been one of the deadliest tropical diseases encountered by humans. Malaria exerts a far more profound influence on progress toward a peaceful life in a given country than have any of the acute epidemic diseases, such as yellow fever. This is because a population stricken with malaria may suffer two negative pressures: acute fatalities from severe malaria, particularly in young children, and long-lasting debilitating symptoms and socio-economic impacts of recurrent and persistent malaria. Here, we present three successive historical stories, stepping stones, the second and third stones having learnt from the previous one, and which was to eventually lead to successful malaria elimination. Each country map tells its own story of change made possible only by an anti-malaria activity.

## Introduction

According to the World Health Organisation (WHO), there were an estimated 228 million cases of malaria worldwide in 2018, with the estimated number of malaria deaths for that year at 405,000. Children aged under 5 years have been the most vulnerable group affected by malaria, and in 2018, they accounted for 67% (272,000) of all malaria deaths worldwide. The WHO African Region carried a disproportionately high share of the global malaria burden, as in 2018, the region was home to 93% of malaria cases and 94% of malaria deaths [1].

This article refers to malaria *management* as a reduction in incidence, prevalence, morbidity or mortality of the disease to a locally acceptable level. Malaria *elimination* is defined as reduction to zero of the incidence of disease or infection in a defined geographical area. ‘Control’ refers to killing the vector (usually with a synthetic insecticide), ‘control’ being a part of vector management. The goal of a vector ‘management’ programme is to prevent disease transmission. All three historical examples discussed in this article practiced vector management.

In 1897, Major Ronald Ross established that malaria was transmitted by anopheline mosquitoes [2]. The situations we address in this article all flow from this seminal discovery. We examine three separate events/stories taking place in severely malarious places where malaria may well have otherwise prevented successful outcomes of the tasks attempted. Events examined are:

1. Construction of the Panama Canal, from the time the USA embarked on it in 1904.
2. Protecting the British Army against malaria in Palestine for six months before the decisive battle against the Turkish Army in 1918.
3. Malaria vector management in Palestine, which began in 1921 and led to durable elimination.

Juxtaposition of these three events that ultimately and significantly changed the political landscape in the world as well as making lasting geographical changes, leads us to pose the question: What results might follow from similar efforts in the remaining seemingly impossible-to-manage malarious areas now and in the future?

## Panama Canal, 1904

For many years, consideration had been given to the construction of a canal to connect the Caribbean Sea and Atlantic Ocean to the Pacific Ocean, to speed up shipping of goods between the east and west coasts of the United States and to Asian ports. A canal across the Isthmus of Panama was an ideal solution but the Isthmus of Panama was also an ideal environment for mosquitoes. Malaria and yellow fever existed there in abundance. French Canal construction efforts began in 1881 but disease decimated the workforce. The French effort eventually collapsed and ceased in 1889 due to financial mis-management and also because the reputation of disease precluded attempts to draw in new workers [3]. Whilst malaria was hospitalising thousands and caused a greater toll in lives than any other one disease, its lower mortality rate did not strike such fear into the populace as did yellow fever.

In 1904, directed by United States Army Surgeon Major W.C. Gorgas, the well-documented construction of the Panama Canal continued. The canal was to eventually extend diagonally across the Isthmus from south-east to north-west, 42 miles from shore to shore. A year earlier, in 1903, Panama granted the United States a concession in perpetuity for a Canal Zone 10 miles wide, 5 miles on either side of the projected Canal line. Whilst Gorgas already had had some experience with the U.S. Army in Cuba in dealing with malaria-carrying *Anopheles* mosquitoes, his main involvement in Cuba had been the introduction of the necessary measures to eliminate the mosquito and thereby successfully handle the yellow fever outbreaks as well as malaria. The military connection in the canal construction was to be maintained with U.S. Army personnel providing engineering expertise. In 1904, Gorgas set out to test the effectiveness of the Ross mosquito theory in the Canal Zone in a large-scale elimination campaign with thousands of men in ‘mosquito brigades’ working year-long to tackle the mosquito population. Other than a full package of malaria management tools including window screening on the barracks, and mandatory prophylactic use of quinine, his method included reduction in malarial mosquitoes through larval source management. Gorgas did this by clearing wide areas of vegetation, draining swamps, ditching, oiling, and larviciding standing water all along or near the proposed route of the canal. When drainage was not possible along the grassy edges of ponds and swamps, oil was added to the water surface, killing mosquito larvae by blocking oxygen intake through their breathing tubes. When oiling was not sufficient, larviciding was attempted. These efforts greatly reduced malaria incidence, and also greatly increased American chances of canal-building success. Yet malaria continued to challenge, albeit to a much lesser degree during the entire construction programme.

Because malaria management was still in its infancy, effective management methods still had to be developed. The work was carried out in a somewhat haphazard manner. A comment in a 1925 report of the Malaria Commission of the League of Nations hinted at probably why the work was conducted this way. The report referred to Ross as one of the greatest malariologists and yet attributed this statement to him:

*"Amateurs are fond of advising that all practical measures should be postponed pending the carrying-out of detailed researches upon the habits of the anophelines ... and so on. In my opinion this is a fundamental mistake. It implies the sacrifice of life and health on a large scale, while researches which may have little real value and which may be continued indefinitely are being attempted. As a matter of fact, the campaigns at ..., Panama, ... were all commenced before the local carriers were definitely incriminated and their habits studied."* [4].

Ross’ reputation was such that it was likely it provided sufficient weight for his suggestions to have been followed. Accordingly, no baseline anti-malaria survey was carried out before the work began. This would have also explained subsequent comments in a 1925 Proceedings of an Antimalarial Advisory Commission Meeting by Dr Samuel Darling, a pathologist specializing in medical zoology and entomology, and colleague of Gorgas during the canal construction. Darling reportedly mentioned:

*“He [Darling] had an unbounded admiration for engineers but … the engineers [engaged in the anti-malaria work] could not [on their own] fulfil all [the roles necessary for] the work. When they had attempted to do so, much unnecessary work had often been carried out: often water had been drained not calling for drainage, and places filled in not requiring to be filled.”* [5].

Apparently, no entomologist was on hand in Panama to direct or guide the indiscriminate work conducted by well-meaning but entomologically uninformed engineers.

**Figure 1:**
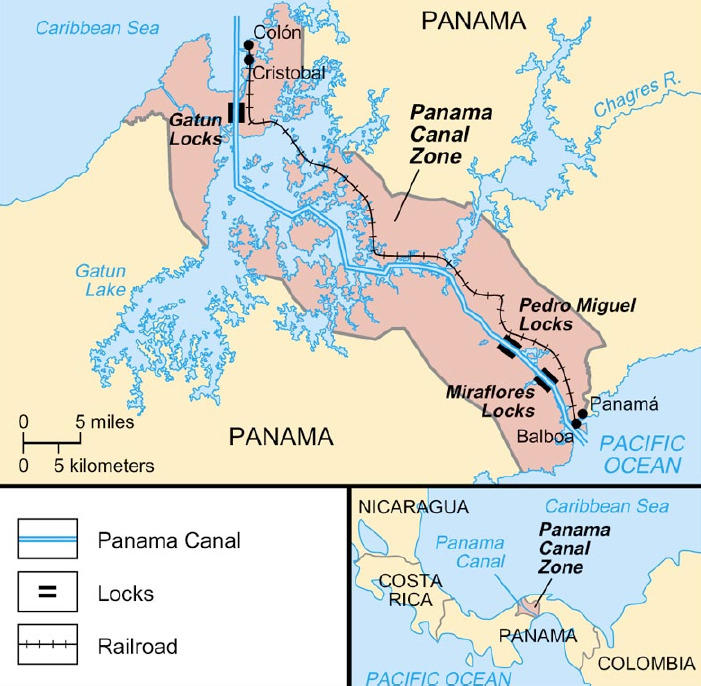
Location of Panama Canal between Pacific and Caribbean/Atlantic [22].

The Panama Canal is still considered the construction miracle of the beginning of the 20th century. At the time, it also was a great demonstration of malaria management based on a mosquito management programme enforced by the military. However, malaria was not eliminated. The disease was simply controlled to the extent that the construction work could be completed. Gorgas was courageous to test the mosquito theory on a scale never before witnessed. There had then never been seen a proven method that rendered severely malarious land relatively safe and usable. The cost was high. Gorgas’ malaria management at the Panama Canal involved thousands of men at high human cost and monetary expense. To remain effective, however, malaria management had to be continuously maintained to ensure mosquito breeding within the managed area did not return. Simply stated, Canal construction was made possible by mosquito management, and the Panama map would henceforth show the Canal connecting the Atlantic and Pacific Oceans (Figure 1).

During Canal construction, malaria management was attempted only in the ten-mile wide strip of the Canal Zone. The reader is reminded that an entomologist was not on hand to direct Gorgas, and malaria management in fact continues there to this day. Guidance to travellers to Panama points out malaria-risk remains, although at least the Panama Canal Zone and certain cities are relatively risk-free.

## Palestine, 1918

**Figure 2:**
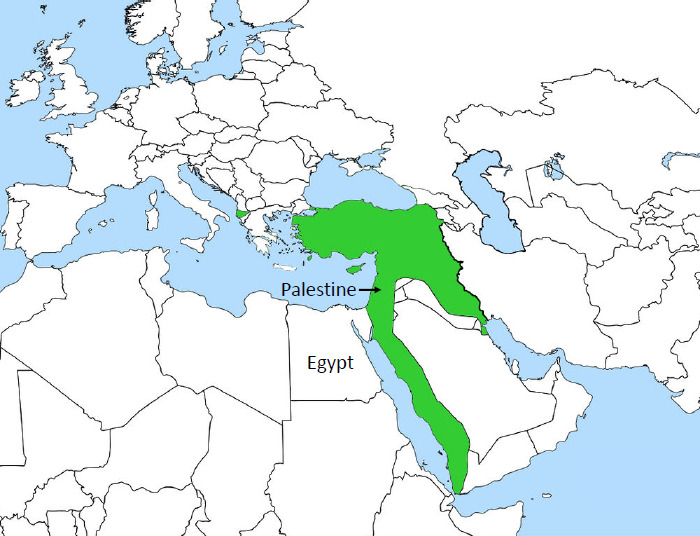
Extent of the Ottoman Empire (green) in 1913 [23].

Palestine was a part of the Ottoman Empire for several centuries (Figure 2). A Century ago, Palestine was notoriously malarious, rendering much of the country either uninhabitable or sparsely populated [6-8].

In 1915, the year after the onset of WWI, the Turkish Army in Palestine attacked British positions on the Suez Canal, Egypt, but was repulsed by the British defenders. In 1916, the British Army invaded Palestine from Egypt, and General Edmund Allenby, an English officer, led the British Army’s Egyptian Expeditionary Force against the Turkish Army in the successful conquest of Palestine in 1917–1918.

It must be remembered that at the beginning of 1918, Gorgas’ earlier anti-malaria work at the Panama Canal was the only example the world had seen of malaria control on an extensive scale. During 1917, Allenby had fought his way northward against the Turkish army and had just captured Jerusalem in December 1917. His army’s lines of supply were becoming over-extended and he needed to pause whilst he regrouped over the following few months, but he then found himself in ‘one of the most highly malarious countries in the world’ [9]. Consideration was accordingly given for withdrawal of his army on this account, but Allenby instead decided to remain where he was, and to take immediate and effective action to protect his troops against the disease. Had Allenby failed to protect his troops, it was later commented, his army would have ‘completely melted away’ [10].

A seemingly-inspired Allenby realised he had to deal with destruction of the mosquito breeding sites in a more precise or targeted fashion than the broad-brush method which had been employed by Gorgas at the Panama Canal, and Allenby chose an entomologist for this purpose. Accordingly, for six months before the final decisive battle in September 1918, anti-malaria works were initiated by Allenby and his entomologist, Major E.E. Austen, to create a ‘healthy’ area for his army and protect his troops in a limited area during the campaign [11].

When planning tactics for his final battle against the Turkish Army, Allenby calculated that he could reasonably count on the British Army lasting no more than ten days after being bitten by infected mosquitoes once his cavalry had crossed the front line, moving northward from the treated British ‘healthy’ area into the malarious area occupied by the Turkish Army. Allenby’s cavalry was ordered to cross the front line and attack on 19 September, and in accordance with his calculations, the British Army began to collapse from malaria on 30 September, but, fortunately for Allenby, only after first having decisively defeated the Turkish army during the previous ten days. Reported, years later about the Palestine campaign, “… a force of 40,500 men … lost 20,427 men almost entirely from malaria [772 killed and wounded, 19,655 debilitated from malaria]” [12].

Suffice it to say that failure to contain malaria would have, in all probability, resulted in failure to defeat the Turkish Army. Of the 100,000 Turkish prisoners-of-war, more than 20,000 had to be admitted to hospital, presumably with malaria as “a very large proportion of the prisoners taken in the firing-line were weak and anaemic as a result of malaria infection” [13].

It is important to appreciate Austen sought merely to control the disease, and the major part of his anti-malaria works involving sometime thousands of Allenby’s troops ceased after Allenby’s final advance began. As the works were not maintained after the advance, malaria returned within weeks.

**Figure 3:**
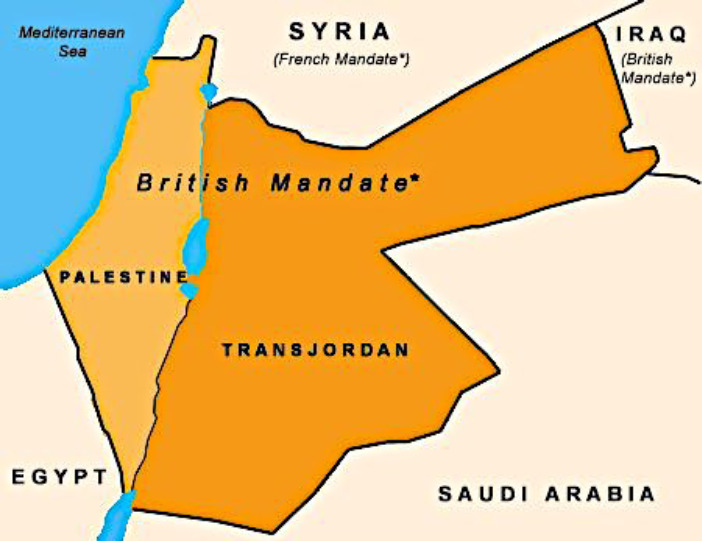
Part of Division of the Ottoman Empire, showing British Mandate 1922. Reprinted with permission of ProCon.org [24].

Successful malaria management directed by the entomologist Austen in 1918 made possible the Turkish Army defeat. Extensive boundary areas of the Middle East were thereafter changed to reflect the division of the former Ottoman Empire (Figure 3). Had Allenby's army 'melted' away due to malaria, history may have shown a different face to the region. These boundary changes were traceable to entomologist Austen’s contribution that enabled the military victory of General Allenby.

## From Mandate to Israel, 1949

### Historical Background

Modern Political Zionism, the movement for Jewish self-determination, arose in the late 19th century as a reaction to anti-Semitic and exclusionary nationalist movements in Europe. Anti-Jewish pogroms in Russia (1881–1884) stimulated growth of Zionism, resulting in formation of pioneering organisations and the first major wave of Jewish immigration to Palestine [14]. Between 1882 and 1914, approximately 75,000 Eastern European Jewish idealists arrived to settle in Palestine, but by 1914, about half this number had died or had left, unable to cope with the severe malarial conditions.

After 19th September 1918, when Allenby began his cavalry charge, the anti-malaria work directed by Austen ceased. Anopheline mosquitoes returned and were again abundant in many formerly ‘healthy’ areas in Palestine. Palestine reverted to its former ‘pre-Allenby/Austen’ state, rendering much of the country again either uninhabitable or sparsely populated.

### The Mandate Period

**Figure 4:**
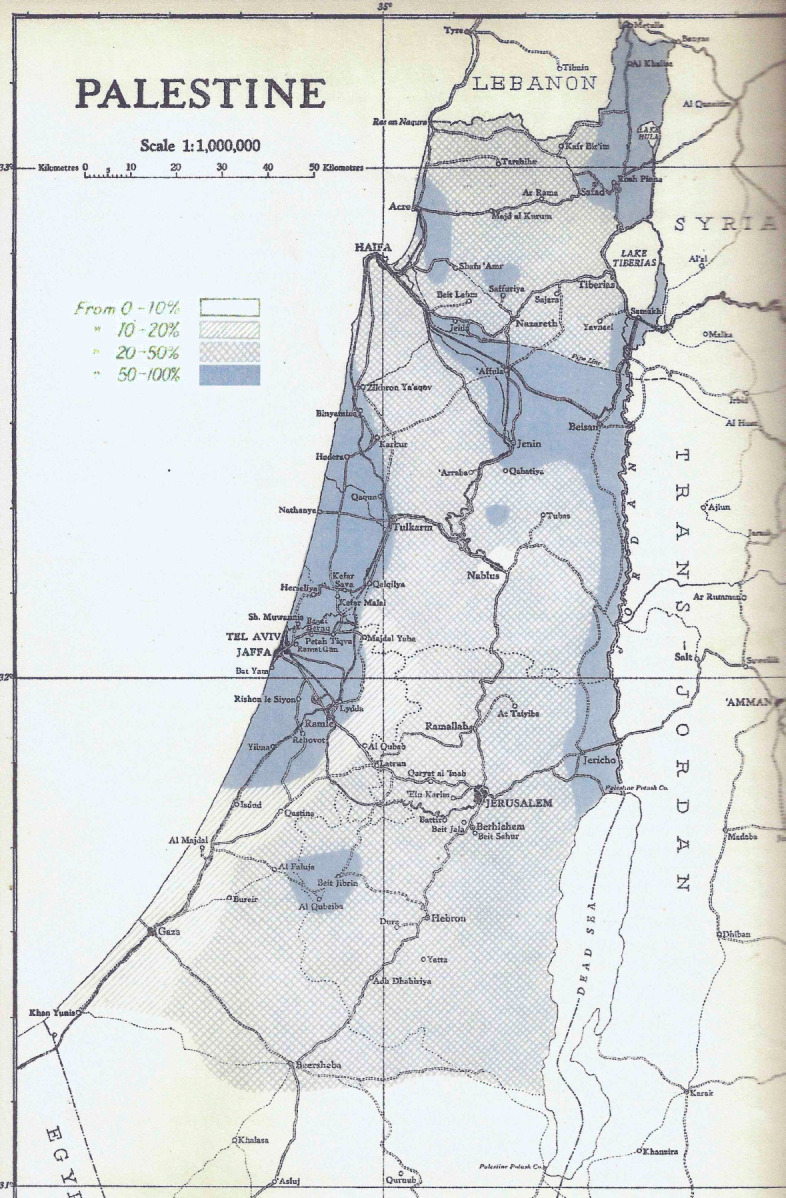
Spleen enlargement rates indicating severe malaria areas in Palestine in 1920 (from [14]).

The Mandate for Palestine was a League of Nations mandate of the territories of Palestine and Transjordan, both of which had been conceded by the Ottoman Empire following WWI. After the 1918 defeat of the Turkish Army by the British Army, the Palestine Mandate, on behalf of the League of Nations, was assigned to the United Kingdom, and operated from 1920 to 1948 by a British civil administration. In 1920, a map of malaria severity in Palestine was drawn (Figure 4) by the British Mandate Department of Health [14]. This map designated the worst areas (dark blue; spleen rates ranging from 50-100%), highly malarious, and the Department of Health even declared some of these areas as uninhabitable. The 1921 Annual Report of the British Mandate’s Department of Health [15] starkly noted that: ‘Malaria stands out as by far the most important disease in Palestine. For centuries it has decimated the population … an effective bar to the development and settlement of large tracts of fertile lands … it assumes epidemic characters in certain areas wiping out the populations of whole villages in a few months’ time … few regions in the country are actually free from it.”

### Kligler’s Malaria Management Method

At the conclusion of WWI, sustainable malaria management by a civil authority in Palestine had been viewed by the British governing authorities as financially impossible, because it would have required considerable manpower for many years. Jewish immigrants seeking to settle in Palestine realised they had to try to undertake steps to eliminate malaria (or malaria transmission) or they would perish. In 1920, Dr. Israel Kligler, an American public health scientist, and an idealistic Zionist Jew, arrived to settle in Palestine, and during 1921–1922, he began what initially was a malaria vector management campaign that was to become the first start anywhere in the world of a successful national malaria-elimination campaign.

In 1920, severely malarious land was of little use because the worldwide experience in malaria management before then had involved thousands of men, as demonstrated by Gorgas at the Panama Canal and by Allenby and Austen in Palestine. Such malaria management efforts had been conducted at enormous human cost and monetary expense. Sustainable and affordable malaria elimination was unknown.

Austen’s and Kligler’s methods were similar in that they were both anti-larval in their approach. The fundamental difference between the two methods was that Austen/Allenby merely needed to control malaria while defeating the Turkish Army, but Kligler needed to permanently eliminate the disease to make the country habitable [6].

In 2017, we emphasised in a previous paper [6] Kligler’s need for education to make malaria elimination durable, pointing out that Kligler noted education was as important as the anti-malaria work itself because unless the population at risk appreciated the ongoing need for vigilance and maintenance as directed by entomologists, malaria would return and the original destruction of the mosquito breeding sites would be of little value [16]. Without the mosquito there can be no malaria.

This was a daunting challenge, to transfer entomological skills to the local population, with its diversity of different cultures and backgrounds, comprising 750,000 people habitually speaking 40 different languages [17] and to transfer these skills in a way that they become naturally and seamlessly shared by elders with future generations.

Whereas Gorgas, Allenby, and Austen could call upon help from thousands of men, Kligler relied on Arab and Jewish co-operation of entire communities—men, women, and children—to assist in the anti-malaria work. This co-operation remained strong, despite attempts to break it by those who opposed it. It was important to Kligler that everyone felt involved. The cooperation endured, and the World Health Organization eventually declared malaria eliminated in Israel in 1967.

When Kligler began anti-malaria work in 1921, he would have described it as malaria management, because malaria elimination as a goal had been considered unlikely. With success of the essential reading of the landscape by the entomologist, and with the culture-based community education and resulting cooperation that Kligler initiated [6], durable malaria elimination became feasible.

### Condition and Location of Land Purchased by Jews

**Figure 5:**
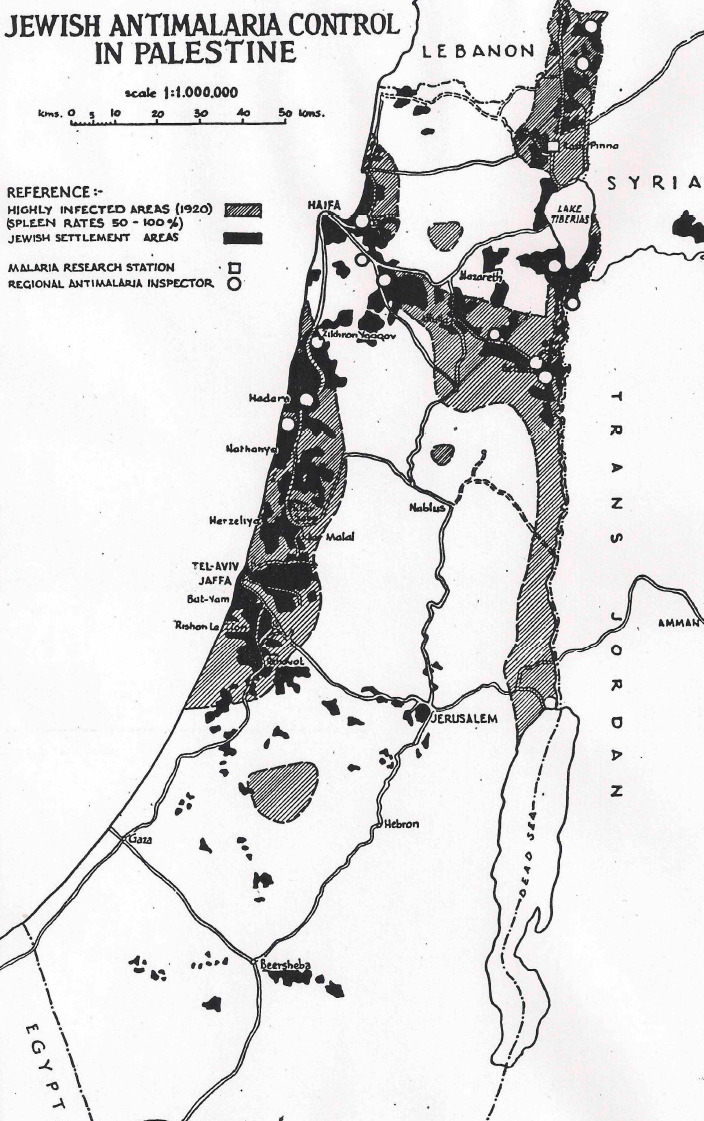
Jewish settlement areas (black) (UN Map 1947) in relation to areas with most severe malaria in 1920.

From 1910 onwards, nearly all lands sold to Jews wishing to settle in Palestine, both before and during the British Mandate period, were located in sparsely populated or uninhabitable, highly malarious areas of the coastal region and the valleys of Palestine [14]. It is not relevant here to consider why Arabs sold or Jews bought land in any particular areas. The fact is that land bought by the Jews was mainly located in the most severely malarious areas, a glimpse of which may be seen in the following examples: The Palestine Department of Health in 1923 [18] noted ‘In … the Valley of Jezreel, from Merchavia to Beisan, … in previous years, there have been severe epidemics of malignant malaria among the newly settled colonists, … [and while there is] … much well-watered and fertile land, [it is] at present lying waste on account of malaria …’. The Palestine Royal Commission in 1937 [19] commented ‘The expenditure [on anti-malaria work] … by the Jews is due to the rapid pace of their colonization and to the fact that they purchased a large amount of land where malaria had been rife for centuries.’; and The British Mandate Palestine Department of Health in its 1941 Review [14] commented (p.5) about rural Jewish immigrants ‘The wave after wave of immigration which has occurred has resulted in the invasion of many sparsely populated and ‘wild’ areas by a people new to malaria.’ The superimposed map (Figure 5) reminds us of the extent to which lands purchased by the Jews were in these previously malarious areas, usually the only lands available to them.

### What Was Achieved?

It is relevant for this paper to stress that by 1941, Kligler’s success in malaria elimination in Palestine was so striking that the British Mandate reported: ‘In a number of areas where intense endemic malaria had resulted in no population for generations, recent [antimalarial] schemes have created large tracts of cultivatable land’ (p. 6) and ‘… very large areas of what is recognised by all as some of the most fertile land in the country have been reclaimed, after centuries of waste,… Many large tracts which until recently meant nothing but death to those venturing into them, have now been reduced into rich and fertile land free from all danger to health’ [14].

### 1949 Israel Armistice Lines

**Figure 6:**
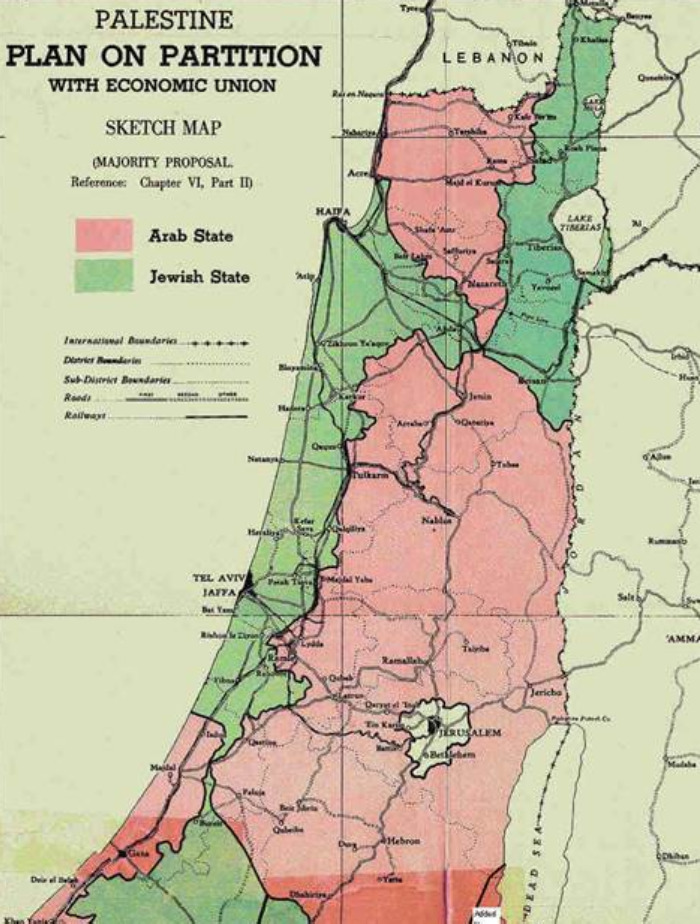
1947 Partition Plan proposed by UN Special Committee on Palestine separating Jewish areas from Arab areas, with Jerusalem remaining internationally administered by UN [25].

From 1929 onwards, the British, the Arabs, and the Jews were involved in periods of violence in Palestine. Britain, unable to control the violence, announced in February 1947 its intention to terminate the Mandate, and referred future matters of Palestine to the United Nations (UN) [20]. The UN recommended a Plan of Partition (Figure 6), which reflected areas where principally the Jews lived and separately where the Arabs lived. The Jews accepted the UN Resolution adopting the Plan, the Arabs rejected it. With the problem unresolved, on 14 May 1948, the British Mandate expired and British forces withdrew from the area. The 1948 Arab-Israeli War began with the invasion of Palestine by neighbouring Arab states on 15 May 1948. The Jews gave battle, their strength being principally concentrated in those areas where they had previously purchased malarious land years before.

**Figure 7:**
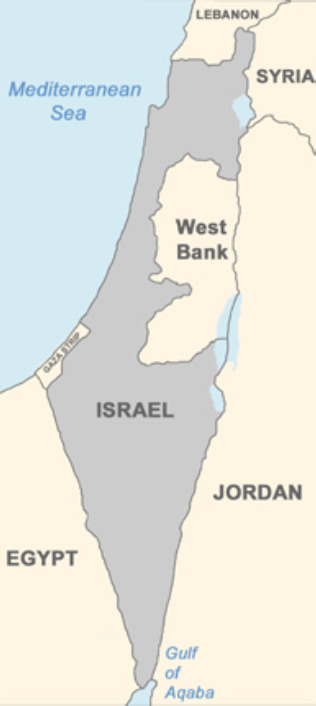
Armistice Lines (1949-1967) reflecting similar shape to 1947 UN Partition Plan, similar to 1920 British Mandate Health Department malaria map (see Figure 4). Reprinted with permission of ProCon.org [26].

The Armistice Lines of 1949 (Figure 7) accordingly reflected a similar shape and pattern to that of the UN Partition Plan, reflecting in turn the ‘dark blue’ malarious areas of the Palestine Department of Health 1920 Map (Figure 4), such 'dark blue' areas including almost the only land that had been available for Jews to purchase. Here revealed is an unanticipated consequence of successful malaria management by an entomologist.

## Entomologists’ Role in Changing World Maps

In the 19th century, when Jewish Zionists began to immigrate to Palestine, then under Ottoman Empire rule, malaria was accepted as a debilitating and, at times, rapidly fatal disease. When medical scientists [2] established that without the female anopheline mosquito, malaria cannot be transmitted to another human, entomologists became the scientists who could rid a community of malaria. Those with the knowledge of an entomologist, such as Austen and Kligler, noted the earlier approach and steps Gorgas took to control malaria, and were there to grasp this key role of ridding a community of this disease. The historical cases discussed in this article formed the basis of our seeing the potential role entomologists can play in changing world maps. These changes in the maps were made possible by defeating malaria: No entomologist, no malaria elimination.

The Ottoman Empire’s rule over the Middle East ended in 1918. Thereafter, the creation of new spheres of influence, new countries, were shown on the maps (Figure 3) of the region. These changes in the maps took place following the defeat of the Turkish army by Allenby, which can be traced back to the vital contribution of Austen, the entomologist. General Allenby had correctly identified malaria control as a priority at the onset of the Palestine campaign, but it was Major Austen, the entomologist, who enabled him to succeed.

The British Mandate’s rule over Palestine ended in 1948. Thereafter, the State of Israel was declared as shown on the map of the 1949 Armistice Lines. Boundaries on the Armistice map to a great extent reflected the concentration of Jews in formerly highly malarious areas in Palestine. Such concentration of Jews in these areas would have been unthinkable when Kligler first began his anti-malaria campaign in 1921/22. Accordingly, the map of the Armistice Lines may be linked with Dr. Kligler, whose entomological experience made possible this unanticipated consequence.

## Conclusion

It is worth remembering that according to WHO statistics, malaria kills more people in twelve days than Ebola has in recorded history, and there is still no absolute defence against malaria. As we have shown, malaria elimination in Palestine first began through use of ‘old’ anti-malaria methods led by entomologists. These ‘old’ methods await re-use, as was successfully done in Mali by one of us (FD). Whilst there now may be many examples of integrated vector management, the weight and priority given to education by Kligler are unlikely to have been demonstrated in such vector management examples. However, an example of innovative education (of which Kligler would certainly have approved) may be seen in Dunkel’s work [21]. Malaria elimination begins with the entomologist in partnership with the at-risk local community that understands malaria is not merely a permanent fact of life. Malaria elimination need be no dream.
